# Choroid and Retinal Nerve Fiber Layer Thickness in Patients with Chronic Obstructive Pulmonary Disease Exacerbation

**DOI:** 10.1155/2018/1201976

**Published:** 2018-07-08

**Authors:** Özkan Kocamış, Duygu Zorlu

**Affiliations:** ^1^Department of Ophthalmology, Faculty of Medicine, Ahi Evran University, Kırşehir, Turkey; ^2^Department of Pulmonary Medicine, Faculty of Medicine, Ahi Evran University, Kırşehir, Turkey

## Abstract

**Purpose:**

We aimed at measuring the choroid and retinal nerve fiber layer thickness with optic coherence tomography (OCT) in patients diagnosed with chronic obstructive pulmonary disease (COPD).

**Methods:**

A total of 60 patients with COPD and 23 healthy controls were evaluated in the scope of this prospective, observational study. COPD patients were divided into two groups as those that were stable and those with an exacerbation based on the Global Initiative for Chronic Obstructive Lung Disease (GOLD) classification. Subfoveal choroid thickness (SFCT) of the patients and the control group was compared by measuring the choroid thickness at points 1000 *µ*m nasal and temporal to the fovea and the mean retinal nerve fiber layer (RNFL) thickness.

**Results:**

The subfoveal choroid thickness of the COPD patients in both the exacerbation and stable groups was found to be statistically significantly thinner than the control group (*p*=0.047 and *p*=0.046, resp.). No statistically significant difference was found between the subfoveal choroid thickness of the patients that were stable and those that had an exacerbation (*p*=0.813). No statistically significant difference was found between the mean RNFL, 1000 *µ*m nasal, or 1000 *µ*m temporal choroid thicknesses of the COPD patients and the control group (*p*=0.263, *p*=0.455, and *p*=0.611, resp.).

**Conclusion:**

Decreased subfoveal choroid thickness was found in the COPD patients both during an exacerbation and in the stable period, when compared to the control group. The mean RNFL thickness was similar in the exacerbation and stable period of the stable COPD patients when compared to the control group. This suggests that ocular findings might be important in terms of COPD morbidity. This trial is registered with www.chictr.org.cn/enIndex.aspx.

## 1. Introduction

Chronic obstructive pulmonary disease (COPD) is a common, preventable, and treatable disease characterized by persistent airflow restriction and respiratory symptoms due to airway and/or alveolar abnormalities that are usually caused by serious exposure to harmful particles or gases [1]. COPD is considered to be a systemic disease that is accompanied by low-grade systemic inflammation causing various systemic effects [2, 3].

The main cause of the inflammation in COPD is exposure to inhaled irritants such as those in cigarettes. These inhaled irritants trigger the production of inflammatory mediators such as C-reactive protein (CRP), interleukins, and fibrinogen and initiate inflammatory events due to the release of leukocytes and thrombocytes from the bone marrow and activation of the circulating leukocytes and vascular endothelial cells [4]. The inflammatory mediators maintain the inflammatory process and cause tissue damage in addition to their various systemic effects.

Ocular complications can also be observed in COPD patient. Ishikawa have found a significant relationship between chronic pulmonary disease including COPD, asthma, lung cancer, and pulmonary fibrosis and preoperative low corneal endothelial cell density [5]. Soler et al. have similarly reported lower endothelial function reserve in COPD patients [6]. Another study has found a relationship between cataracts and COPD exacerbations [7].

The retinal and choroidal microvasculatures are complex vascular systems that can become involved in many systemic disorders [8]. A functionally normal choroidal vasculature is essential for retinal function; thinning of the choroid and loss of the vascular tissues often leads to photoreceptor damage and vascular dysfunction [9].

Hypoxia and systemic inflammation are thought to involve fine ocular structures, such as the choroid, macula, retinal nerve fiber layer (RNFL), and retinal vascular vessels. These can all be assessed quantitatively and qualitatively using spectral domain OCT (SD-OCT) [10].

Enhanced depth imaging OCT (EDI-OCT) is a relatively new technique that uses light with a longer wavelength as it is more effective for choroidal scanning. It provides valuable data about the choroidal morphology in both healthy subjects and in those with various diseases [11–17].

We aimed at evaluating the macular choroid thickness in COPD patients by using EDI-OCT in this study.

## 2. Materials and Methods

This prospective and observational study was approved by the ethics committee of our institution (2017-17/207). A total of 83 patients being followed up at the Ahi Evran University's Department of Pulmonary Disease and consisting of 60 patients with COPD and 23 healthy controls of similar age and gender were evaluated within the scope of the study. The study protocol was based on the Helsinki Declaration principles. Pulmonary function tests were performed on the patients with COPD. Pulse oximetry from the fingertip was used to determine hypoxic conditions. Pulse oximetry measurements were <97%, and we did not perform blood gas analysis, an invasive method, as the patients were known to be hypoxemic. We classified the COPD patients into two with 30 patients in the exacerbation period and 30 patients in the stable period. The definition of exacerbation in GOLD 2017 is “acute worsening in addition to the symptoms, resulting in additional treatment requirement” [1]. The classification of exacerbation severity was based on the treatment options used as mild, moderate, and severe in the GOLD 2017 report [1, 18]:

- Mild: Patient treated only with short-acting bronchodilators (SAB).
- Moderate: Patient treated with antibiotics and/or oral corticosteroids in addition to SAB.
- Severe: Patient presents to the emergency service or is admitted to the hospital. Severe exacerbation can also coexist with acute respiratory failure.

COPD patients during the exacerbation period consisted of patients with moderate exacerbation severity not requiring hospitalization. The medication used by the COPD patients consisted of *β*2 agonists (salmuterol and formoterol), anticholinergics (tiotropium), oral antibiotics (sefuroxime and ofloxacin), and oral corticosteroids (dexamethasone). A total of 23 subjects who did not smoke and whose pulse oximetry measurements were 97% or higher were identified as the control group.

The inclusion criteria were a visual acuity of 0.8 or better, a spherical refractive error less than 3 *D* and astigmatic refractive error less than 3 *D*, intraocular pressure below 21 mmHg, and axial length less than 24 mm.

Patients with diabetes mellitus (DM), hypertension (HT), cardiac failure, those with a history of ocular surgery, and patients with age-related macular degeneration or glaucoma were excluded from the study.

### 2.1. Ocular Examinations

**Figure 1:**
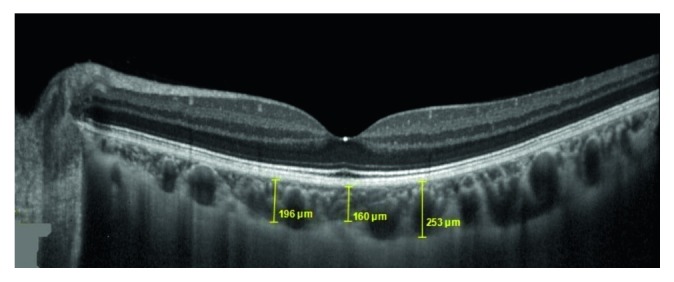
Choroidal measurements of the patients diagnosed with COPD.

The left eyes were included in the study. All subjects underwent standard ophthalmologic examination with a visual acuity test using the Snellen chart, biomicroscopy, noncontact tonometer intraocular pressure measurement, axial length measurement, fundus examination, and SD-OCT (Spectralis, Heidelberg, Germany) measurements. The subfoveal choroidal thickness (SFCT) and the choroidal thickness 1000 *µ*m nasal and 1000 *µ*m temporal to the subfoveal were measured with EDI-OCT. Choroid thickness was measured from the inner surface of the sclera to the outer portion of the hyperreflective line corresponding to the retinal pigment epithelium (Figure 1). The chorioscleral interface was clearly displayed for all measurements. Peripapillary retinal nerve fiber layer (RNFL) thickness was also measured by SD-OCT. The global thickness was recorded as the RNFL thickness.

### 2.2. Statistical Analysis

Mean, standard deviation, median, the lowest, the highest, frequency, and ratio values were used in the descriptive statistics of the data. The distribution of the variables was measured with the Kolmogorov–Smirnov test. Independent samples *t*-test and the Mann–Whitney *U* test were used in the analysis of the quantitative independent data. The SPSS 22.0 program was used for the analyses.

## 3. Results

The mean age of the COPD patients evaluated within the scope of the study was 64.1 ± 7.3 years in the exacerbation group, 64.4 ± 8.6 years in the stable group, and 65.8 ± 7.1 years in the control group. No statistically significant difference was present between the FEV1 and FEV1/FVC values of those in the exacerbation and stable groups (*p*=0.054 and *p*=0.092, resp.). The oxygen saturations of the patients in the exacerbation group were statistically significantly lower than those in the stable group (*p*=0.009; Table 1). No statistically significant difference was present between the mean RSLT, 1000 *µ*m nasal, and 1000 *µ*m temporal choroid thicknesses of the COPD patients and the control group (*p*=0.263, *p*=0.455, and *p*=0.611, resp.). The subfoveal choroid thickness of the patients in both the exacerbation and stable COPD groups was statistically significantly lower than in the control group (*p*=0.047 and *p*=0.046, resp.). No statistically significant difference was found between the subfoveal choroid thickness of the exacerbation group and the stable group (*p*=0.813; Table 2).

## 4. Discussion

The choroid is a complex vascular network that supplies the retinal pigment epithelium and outer retina layers. It also provides thermal stability for the ocular tissues, removes ocular waste, and plays a role in uveoscleral aqueous drainage and intraocular pressure regulation [19, 20].

We found significantly lower subfoveal choroid thickness in COPD patients in the exacerbation and stable groups compared to the control group in our study. No significant difference was found in the choroid thickness 1000 *µ* nasal and temporal to the fovea in the COPD groups compared to the control group. No significant difference was found between the mean RNFL thicknesses of the COPD patients in the exacerbation and stable groups.

Many factors can influence choroid thickness. The value decreases with age [21] but increases in acromegaly and diabetes [22, 23]. The choroid thickness reflects the choroid circulation and can be influenced by the systemic blood pressure, intraocular pressure, mean ocular perfusion pressure, nitric oxide, catecholamines, and vascular autoregulation [24]. The levels of endothelin-1 (ET-1), a potent vasoconstrictor, have been shown to be increased in the plasma, sputum, and urine samples of COPD patients compared with those in controls [25, 26].

A dose-dependent vasoconstrictor effect of ET-1 has been reported by Polak et al. and mainly affects retinal microvessels [27]. Increased ET-1 levels in the plasma are thought to increase resistance to ocular artery blood flow in COPD patients through vasospasm. Such increased levels could develop as a result of chronic hypoxia. Nikolaou et al. have reported increased blood ET-1 levels during nocturnal oxyhemoglobin desaturation in these patients [28].

Chronic hypoxia and hypercapnia may affect the retinal and choroidal blood flow in COPD patients. In this regard, some color Doppler ultrasonography studies have demonstrated impaired retrobulbar hemodynamics, reduced retrobulbar blood flow, and increased resistance to blood flow in both the retinal and choroidal circulation in COPD patients [29–31].

Ozcimen et al. evaluated the peripapillary choroid thicknesses in COPD patients and found thinning in all segments; however, statistically significant thinning was only present in the inferior segment [9]. Ugurlu et al. found no statistically significant difference between COPD patients and the control group in terms of subfoveal choroid thickness [32] (*p*=0.84). Gok et al. showed that macular choroid thicknesses in all COPD stages were lower than in the control group at all locations, but no statistically significant difference was present [8]. The subfoveal choroid thickness was statistically significantly lower in COPD patients in the exacerbation and stable groups in our study but no difference was present in other locations.

The chronic systemic inflammation and hypoxia in COPD can cause oxidative stress substances to appear and lead to a disturbed oxidant-antioxidant balance [33, 34], resulting in axonal loss and ganglion cell death [35–37]. The end result is decreased RNFL thickness.

Ugurlu et al. reported lower RNFL thickness in all segments in COPD patients but a significant thinning was only present in the inferior segment [32]. In contrast to our study, Ozcimen et al. found the mean RNFL thickness in patients with COPD to be significantly lower [9]. Gok et al. found significantly lower values for both mean and nasal segment RSLT thickness in patients with COPD [8]. We evaluated the mean RNFL thickness and did not perform segmentation.

## 5. Conclusions

Our study has some limitations. First of all, the chorioscleral border was defined manually, and the accuracy of this process was not evaluated. Secondly, the number of COPD patients and especially of patients in the exacerbation group was relatively low.

The fact that the COPD patients showed lower choroid thickness in both the stable and exacerbation groups indicates that it may be possible to use this finding as an indicator of COPD morbidity.

## Figures and Tables

**Table 1:** Clinical and demographic characteristics of the COPD and control groups.

	Group A (*n*=30)	Group B (*n*=30)	Control (*n*=23)	*p*
Age (y)	64.1 ± 7.3	64.4 ± 8.6	65.8 ± 7.1	0.073^A^
FEV1/FVC	63.9 ± 14.8	71.1 ± 13.1	90.2 ± 4.2	0.092^m^
FEV1	1.6 ± 0.7	2.0 ± 0.8	2.2 ± 0.5	0.054^m^
O_2_	89.3 ± 8.5	94.2 ± 4.4	98 ± 0.9	0.009^m^

Group A: COPD at the exacerbation period; Group B: COPD at the stable period; ^A^ANOVA; ^m^Mann–Whitney *U* test; FEV1: forced expiratory volume; FVC: forced vital capacity; O_2_: oxygen saturation; y: year.

**Table 2:** Choroid and RNFL thickness values of the COPD and control groups.

	Group A (*n*=30)	Group B (*n*=30)	Control (*n*=23)	*p*	*p* ^*∗*^	*p* ^*∗∗*^	*p* ^*∗∗∗*^
SFCT	228.3 ± 76.2	224.3 ± 78.7	269.0 ± 86.7	0.016^K^	0.813	0.047	0.046
N	207.6 ± 82.1	208.3 ± 85.3	216.9 ± 64.3	0.455^K^	0.976	0.170	0.414
T	204.6 ± 66.9	213.6 ± 67.1	218.9 ± 66.9	0.611^K^	0.487	0.389	0.596
RSLT	103.0 ± 11.4	98.3 ± 11.3	100.1 ± 9.4	0.263^K^	0.113	0.265	0.767

Group A: COPD at the exacerbation period; Group B: COPD at the stable period; SFCT: subfoveal choroidal thickness; N: nasal choroid; T: temporal choroid; RNFL: retinal nerve fiber layer; ^K^Kruskal–Wallis; ^*∗*^*p*: between Groups A and B; ^*∗∗*^*p*: between Group A and controls; ^*∗∗∗*^*p*: between Group B and controls.

## Data Availability

The data used to support the findings of this study are available from the corresponding author upon request.
